# Is relief the most common reaction to abortion? Self-assessed intensity of emotions attributed to abortion in a national sample of women aged 41 to 45

**DOI:** 10.12688/f1000research.162063.1

**Published:** 2025-02-26

**Authors:** David Reardon

**Affiliations:** 1 Elliot Institute, Gulf Breeze, FL, 32563, USA; 2 Charlotte Lozier Institute, Arlington, Virginia, USA

**Keywords:** abortion, pregnancy loss, miscarriage, mental health, emotional reactions to pregnancy loss

## Abstract

**Background:**

It is widely reported that the most prominent emotion following abortions is relief. This claim primarily rests on two studies of abortion clinic patients which had methodological and self-censure bias. Other studies have indicated that negative emotions are more common than positive emotions. The objective of this study is to obtain self-assessed data on the intensity of emotional responses to abortion and pregnancy loss in a random national sample.

**Methods:**

Using visual analog scales, a random sample of 1,925 women aged 41 to 45 completed a survey in which respondents rated the degree to which they experienced emotional responses to their first abortion or natural pregnancy loss. The emotions assessed included relief, grief, depression, anxiety, guilt, emptiness, anger, regret, shame, unforgiveness of self, uncontrollable weeping, frequent thoughts of the child they could have had, and difficulty completing the grief process. Women were categorized into five groups based on pregnancy outcomes, and four abortion decision types: Wanted, Inconsistent, Unwanted, or Coerced.

**Results:**

Among women with a history of abortion (n=409), negative emotions were reported more intensely than relief. Relief was the predominant emotion only among the 29.8% of women whose abortions were freely wanted and consistent with their own values and preferences. For all other groups, relief was low and negative emotions were more prominent. Emotions following natural pregnancy losses were similar to those following abortion, but less severe following wanted abortions and more severe following coerced abortions.

**Conclusions:**

Relief is only common after freely wanted abortions. Most abortions are inconsistent or contrary to women’s own values. In these cases, strong negative emotions are far more dominant than relief. These results should inform pre-abortion screening, counseling supportive of women’s own values and preferences, and mental health support post-abortion.

## Introduction

Planned Parenthood, the largest abortion provider in America, claims that “Over 95% of people who have had abortions say that they mostly felt relief after their abortion.” ^1^ But this claim conflicts with a substantial body of literature showing abortion is associated with higher rates of mental illness and widespread negative emotions. ^2– 10^ It is important for those considering abortion, and those providing medical advice, to have accurate information about emotions which commonly follow an abortion.

A literature search identified two studies that appear to be the primary basis for the claim that relief is the most common reaction to an abortion.

First, there is a longitudinal study of 442 women recruited at abortion clinics in 1993 which found that women reported “more relief than negative emotions” both one hour after their abortions and at a subsequent interview two years later. ^11^ But notably, the researchers also found that over the two-year period relief and positive emotions declined and negative emotions had significantly increased. This study had numerous limitations. First, the list of negative emotions assessed included only six items. They were “sad,” “blue,” “low,” “disappointed,” “guilty,” and “feelings of loss.” Second, the investigators did not disclose how each of these six items individually compared to relief. Therefore, the average of six variables compared to a single variable (relief ) may have concealed the prominence of single negative emotions (such as guilt) relative to relief. Fourth, three of the negative emotions (“sad” “blue” and “low”) would appear to be measures of the same affect, depriving the scale of breadth. Fifth, while sadness, guilt, and feelings of loss have been widely reported in qualitative studies, disappointment has not. ^12^ If “disappointed” is a weak or infrequently experienced emotion, inclusion of this item would tend to suppress the aggregate score for negative emotions. As a result, it is quite possible that the average score of guilty, for example, was greater than that for relief, but this could not be determined with reporting of only the aggregate average of the six negative emotions. Notably, both requests for clarification of these concerns and requests for the anonymized data for reanalysis were refused. ^12^ The sixth, and most important limitation of this study is that the percentage of invited participants (n = 1117) dropped from 74.9% participation in a pre-abortion interview to 52.3% at the one-month post-abortion interview, and only 37.6% at the two-year post abortion interview. This high drop-out rate introduces the risk of substantial self-censure bias. If the women who experienced the most negative reactions were most prone to dropping out, the longitudinal analysis has less merit and might even be misleading. Finally, for unexplained reasons, women whose pregnancies were intended or were seeking second trimester abortions were excluded from the study. This introduced methodological sampling bias.

Second, there is a longitudinal survey of 667 women recruited at abortion clinics which reported that “Relief was the most commonly felt emotion at all times over 5 years post-abortion”. ^13^ In this assessment, only four negative emotions were considered: guilt, sadness, regret, anger. As in the prior study, relief declined over time, but the authors’ analyses indicated the likelihood that it remained above the average scores for each of the negative emotions over the five years investigated. The major shortcomings of this study include both methodological and self-censure bias. Invitations to participate were at the discretion of abortion clinic counselors and were therefore not random. In addition, women seeking abortions when there was a diagnosis of fetal malformation were automatically excluded. Most seriously, despite an inducement of $50 per interview, only 31% of those who were invited participated in the first interview and participation declined to 17% participation at the last interview, five years later.

Non-participation and concealment of abortions are common problems. In national surveys, it is typical that the number of abortions women admit having had are only half of the expected rate based on national abortion statistics. ^14, 15^ Studies conducted with the cooperation of abortion clinic staff have revealed reasonably high rates of participation can be achieved in pre-abortion interviews ^11, 16^ but there are very high dropout rates for post-abortion interviews after subjects have left the abortion provider. Studies examining attrition associated with pre-abortion interview data reveal that those who dropout are more likely to fit into the categories of those who anticipate and/or experience the most negative emotional and mental health reactions self-attributed to their abortions. ^7, 17, 18^ This is most likely due to a fear that such interviews may arouse, or even intensify, negative feelings. ^12^ This is consistent with previous research which has shown that women reporting both a history of abortion and substance abuse reported higher levels of stress in completing a survey touching on these topics. ^19^

Regarding the women who do experience more negative emotions, a number of other studies have reported that those who feel pressured to abort or otherwise feel most conflicted about their own decision are most likely to report less relief and more negative outcomes. ^11, 20– 26^ One study, focused on women who participating in post-abortion healing programs, found that only 38% reported having felt strong feelings of relief following their abortions. ^5^ Of those reporting relief, 60% stated these feelings of relief dissipated within weeks and only 25% reported they persisted for years. More common reactions were guilt (93%), grief (85%), regret (85%), anger (81%), and others. ^5^ This predominance of negative reactions in this investigation was clearly due to this study’s exclusive reliance on women seeking post-abortion emotional support. Despite this limitation, it underscores the fact that underrepresentation of this subset of women in abortion clinic-based surveys, whether due to methodological or self-censure bias, would most likely lead to dramatic understatement of negative emotions and an overstatement of relief.

Planned Parenthood has also reported that “Terminating a wanted pregnancy can be associated with negative psychological experiences comparable to those associated with stillbirth or death of a newborn“. ^27^ This is consistent with the American Psychological Association’s 2008 literature review that found that women who feel pressured to abort, and/or are more emotionally attached to their pregnancies, and/or are ambivalent about their abortion decisions, are at greater risk of more negative reactions and less relief. ^12, 28^ There is evidence that the majority of women seeking abortions fall within one or more of these higher risk categories. ^6, 7, 12^ Therefore, it is reasonable to expect the degrees of feelings of relief to vary significantly by abortion decision types. It is also reasonable to expect that differences in abortion decision types may make the abortion experience more or less similar to that of a miscarriage or other natural loss.

Given these observations, this investigation is an attempt to measure the degree to which relief and a broad variety of negative emotions are self-reported by a random sample of women, to investigate any self-selection bias and the degree of stress reported in participating in such research, and to identify differences across different abortion decision types in comparison to the degree of feelings reported by women who experienced natural pregnancy losses.

### Hypotheses

• Null Hypothesis (H1): Relief is the most common feeling women experience after an abortion.
• Alternative Hypothesis (H2): Following an induced abortion, negative feelings are far more common than relief.
• Alternative Hypothesis (H3): Negative feelings following an induced abortion are similar to those following a natural pregnancy loss.
• Alternative Hypothesis (H4): The degree and type of negative feelings following an induced abortion will vary according to the degree the abortion decision type includes conflict with the woman’s own values and preferences.
• Alternative Hypothesis (H5): More negative feelings about an abortion will be associated with greater stress when participating in surveys about the abortion experience.

## Methods

The questionnaire was developed with experts with experience in abortion and mental health research.

The survey was hosted by LimeSurvey.org, utilizing the open source web survey tool, LimeSurvey v6.10.4, and distributed using the services of Cint.com, one of the world’s largest digital marketing and social sciences survey research firms. Cint has over 28 million U.S. residents in their survey panels. Cint panelists voluntarily participate in surveys using their personal electronic devices in exchange for small incentives, which, for this study, were valued at under $2 per completed survey. Panelists electronically signed a consent form including the disclosure that the survey might include “sensitive” subject matter. To avoid introducing any survey selection bias, no additional description of the subject matter was provided. A random sample of Cint panelists pre-identified as female residents of the United States, aged 41 to 45, were invited to complete the survey until 1,500 surveys were completed by women reporting at least one pregnancy outcome. The selected age range minimized the confounding effects of age and focused the sample on those whose history of pregnancies was mostly completed. The survey was distributed electronically, and data collection occurred over three days in July 2024.

### Survey design and stigma reduction questions

The first page of the questionnaire asked about age and gender to qualify respondents. The second page asked respondents to identify any mental illnesses for which they had ever been diagnosed. This served as the first response bias test regarding respondent’s willingness to share sensitive information.

Prior to asking about reproductive histories, three abortion stigma reduction questions were presented to respondents. These were developed in light of the fact that national surveys of women have typically disclosed only half of the expected number of abortions compared to national statistics. ^14, 15^ It is hypothesized that concealment of abortion histories are most likely due to feelings of shame, guilt, secrecy or similar feelings that disincline some women to share their abortion histories. To diffuse such reluctance, our survey included three stigma reduction questions, prior to a direct inquiry about whether the respondent had ever had an abortion. The first stigma reduction question was: “Some women feel pressured into abortions by other people (like a male partner) or by circumstances (like poverty). Do you believe the problem of women feeling pressured to have abortions is very rare, uncommon, somewhat common, or very common?” The second was, “How many people do you know who felt pressured to have abortions?” The third asked, “Even if you did not go through with it, have you ever felt pressured to consider an abortion?”

This approach was designed to incrementally prepare respondents to consider disclosing past abortions by first allowing them to consider and share whether they had experienced pressure to have an abortion from other people or circumstances. Since this is a major risk factor for more negative feelings, ^6^ this question invites those who might otherwise be initially inclined to conceal their abortions to acknowledge that portion of their experience which was a feeling of facing pressures to choose an abortion. They are then invited to consider if and how many other women they know who may have had a similar experience. Then, and only then, are they directly asked if they themselves ever felt pressured to consider an abortion, even if they had not gone through with it. For those who felt such pressures, an affirmative response to the last question is at least half a step toward revealing their own abortion experience. In previous studies using these abortion stigma reduction questions, the number of women reporting a history of abortion closely matched the national estimate for abortion rates. ^6, 7^

Respondents were then asked to identify the number of times they had (a) given birth to a live born child, (b) had given birth to a child that was the result of an “unplanned, mistimed, unwanted, or otherwise difficult pregnancy” (problematic pregnancy), (c) had a “miscarriage, still birth, or other pregnancy loss”, or (d) had an induced abortion.

From this reported pregnancy history women were divided by a program algorithm into one of five groups, by order of priority, (a) induced abortion group, (b) natural pregnancy loss group, (c) problematic pregnancy group, (d) live birth group, and (e) never pregnant group. Given the programmatic priority structure, women in the abortions group may also have had one or more other pregnancy outcomes. But women in the natural pregnancy loss group would not have reported any abortions but could have had live births and/or problematic pregnancies.

**Figure 1.**
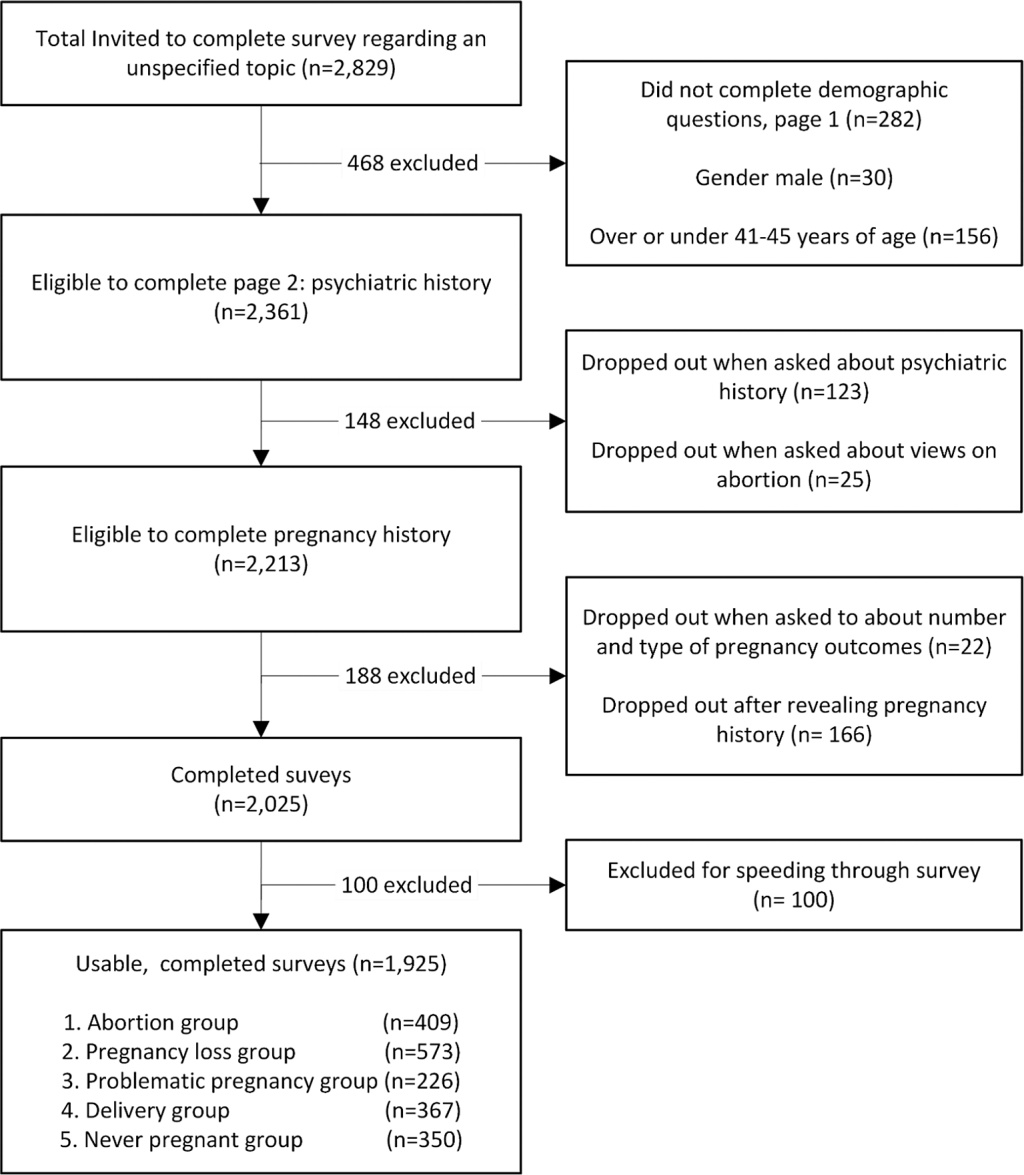
Survey population.

The study population is shown in Figure 1. Respondents who failed to complete all survey questions or fell outside the specified gender and age criteria were excluded from analysis. Of the 2,361 participants who completed the first two demographic questions, 123 (5.2%) dropped out when presented with the psychiatric history section. An additional 25 (1.1%) dropped out when presented with the abortion stigma reduction questions followed by another 22 (1.0%) who discontinued participation when asked to number all of their own pregnancy outcomes. Another 166 (7.0%) withdrew after reporting their pregnancy histories but before completing the full survey. In consultation with Cint, since the survey was designed to take approximately five to seven minutes for respondents to complete, 100 respondents (4.6%) were excluded for finishing in under four minutes to eliminate “speedsters.” This is a subset of persons signing up to complete Cint distributed surveys who randomly respond to questions as quickly as possible in the hope of earning Cint credits without actually reading or attentively responding to the survey items.

### Variables used

Respondents who had a history of abortion or natural pregnancy loss were asked to rate, on visual analog sliding scales, the degree to which their first abortion, or first pregnancy loss, contributed to each emotional reaction. The scales were relief, grief, depression, anxiety, guilt, emptiness, anger, regret, shame, unforgiveness of self, uncontrollable weeping, “frequent thoughts of the child I could have had,” and “difficulty completing the grief process.” The left most end of each sliding scale read “Not at all” and the right most end “Very much.” The starting position of the button slid along the slider line was in the middle of the scale. After movement of the slider, and acceptance of all responses entered by the subject on the displayed survey page, the position of the slider was electronically and proportionately coded on a 101-point scale, from 0 (Not at all) to 100 (Very much). A visual analog scale was also used to encode the degree of disagreement (“Not at all true”) to agreement (“Very true”) with the last item in the survey, “Completing this survey has increased feelings of stress.”

For women who reported induced abortions, an additional categorical question was asked: “Which best describes your abortion decision?” Respondents were presented with four possible answers: “Wanted and consistent with my values and preferences,” (Wanted), “Accepted but inconsistent with my values or preferences” (Inconsistent), “Unwanted and contrary to my values and preferences” (Unwanted) or “Coerced and contrary to my values and preferences” (Coerced). For parametric purposes, these were scored from 1 to 4 in the order presented above.

### Statistical analyses

For all analyses, a p-value less than 0.05 was considered statistically significant. Abnormally distributed continuous variables are reported with medians and interquartile ranges (IQRs). Differences between groups were tested for significant differences using Mann-Whitney test statistic (U) and Hedge’s g for effect size. Differences between emotions were tested using Wilcoxon signed rank test statistic (W) and effect size was calculated using rank-biserial correlation (RBC) statistics. In each emotion comparison, Relief was the reference emotion and paired with each of the other emotions in separate Wilcoxon signed rank tests. When interpreting effect size, for both Hedge’s g and RBC, less than 0.3 was considered a small effect, between 0.3 and 0.5 a medium effect, and greater than 0.5 a large effect. A multiple linear regression model was used to test the effects of emotions reported after an abortion and abortion decision types on levels of stress experienced in completing a post-abortion survey. Analyses were conducted using JASP 0.19.

## Results

Our final study population consisted of 1,925 completed surveys, of whom 409 (21.2%) reported one or more abortions which is only 10.5% lower than estimated 23.7% lifetime abortion rate estimated for women between 41-45 years of age by the Guttmacher Institute. ^29^ Another 573 (29.8%) respondents reported a natural pregnancy loss and did not also have a history of abortion. Within the abortion group, 56.3% (n=230) also reported having one or more natural pregnancy losses. Among women who had abortions, 122 (29.9%) described their abortions as wanted and consistent with their values and preferences (Wanted), 145 (35.5%) described it as accepted but inconsistent with their values and preferences (Inconsistent), 90 (22.0%) described it as unwanted and contrary to their values and preferences (Unwanted), and 52 (12.7%) described it as coerced and contrary to their values and preferences (Coerced).

Table 1 shows descriptive statistics and Mann Whitney test results comparing the intensity of emotions reported by women in the abortion group compared to women in the pregnancy loss group. These findings found that while there are statistically significant differences in the degree some emotions are felt by each group, the low Hedge’s g score (all below 0.3) indicate the degree of differences are small. Specifically, emotions that were significantly higher (p < 0.05) following abortion included guilt, shame, regret, unforgiveness of self. In contrast, following a pregnancy loss, feelings of emptiness, weeping, grief, anger, anxiety, and child related thoughts were significantly higher, but with low effect sizes (g < 0.3). There were no statistically significant differences regarding depression and difficulty completing the grief process. These findings suggest rejection of the null hypothesis (H1) which posited that relief is the most common reaction following an abortion and support H2 which posited that negative emotions are more common than relief post-abortion. These results also partially support H3, which posited that there are similarities in emotions following abortion and natural pregnancy losses.

**Table 1.** IQR, quartiles and Mann-Whitney U Signed-Rank Test for emotions following abortion and pregnancy loss with emotions sorted by highest median for abortion.

		IQR	25th percentile	50th percentile (median)	75th percentile	U	p	Hedge’s g
Guilt	abortion	49	50	83	99	137130	**<.001**	0.17
	pregnancy loss	68	26	69	94			
Grief	abortion	68	30	75	98	108283	**0.04**	-0.08
	pregnancy loss	51	48	78	99			
Depression	abortion	67	31	72	98	110880	0.15	-0.05
	pregnancy loss	53	45	76	98			
Shame	abortion	69	26	71	95	145376	**<.001**	0.24
	pregnancy loss	74	3	35	77			
Thoughts of child	abortion	68	23	69	91	105967	**0.01**	-0.1
	pregnancy loss	62	36	73	98			
Regret	abortion	75	19	67	94	132625	**<.001**	0.13
	pregnancy loss	71	11	51	82			
Anxiety	abortion	67	27	67	94	107882	**0.03**	-0.08
	pregnancy loss	57	40	73	97			
Unforgiveness	abortion	76	19	66	95	131284	**0.001**	0.12
	pregnancy loss	77	6	50	83			
Anger	abortion	70	15	62	85	108323	**0.04**	-0.08
	pregnancy loss	64	27	67	91			
Emptiness	abortion	66	21	62	87	96077	**<.001**	-0.18
	pregnancy loss	51	47	74	98			
Relief	abortion	62	20	56	82	152758	**<.001**	0.30
	pregnancy loss	62	1	24	63			
Difficult grieving	abortion	79	3	52	82	119091	0.66	0.02
	pregnancy loss	74	6	48	80			
Weeping	abortion	80	5	51	85	104292	**0.003**	-0.11
	pregnancy loss	70	21	64	91			

*Note.* For the Mann-Whitney test, effect size is given by the rank biserial correlation.

Table 2 disaggregates the results for women who had abortions and shows the descriptive statistics and Mann Whitney test results for each abortion decision type, using Wanted decisions as the reference group. These results show significantly more intense negative emotions (and less relief ) for each emotion examined (p < 0.001) for the Inconsistent, Unwanted, and Coerced abortion decision types compared to the Wanted decision type. The effect size was moderate (g > 0.3) to strong (g > 0.5) in nearly every comparison, with the greatest differences observed between freely wanted abortions and those respondents described as coerced. Notably, IQR scores indicate that responses for the middle 50% of the Wanted group are widely spread out (IQR > 50) while responses for Coerced were typically more tightly grouped (IQR < 30), indicating more uniformity in the latter group. These results strongly support hypothesis H4, which posited that the degree and type of negative reactions would vary according to abortion decision types.

**Table 2.** IQR and quartiles and Mann-Whitney U test scores each emotion scale disaggregated by abortion decision type.

		IQR	25th percentile	50th percentile	75th percentile	U	p	Hedge’s g
Guilt	Wanted	84.75	2.25	63.5	87	ref	ref	ref
	Inconsistent	34	66	85	100	12135.5	**<.001**	0.37
	Unwanted	37.75	62.25	88	100	7491	**<.001**	0.36
	Coerced	24.25	75.75	98	100	4701	**<.001**	0.48
Grief	Wanted	80.75	0.25	51	81	ref	ref	ref
	Inconsistent	40	59	76	99	12128.5	**<.001**	0.37
	Unwanted	51.25	46.75	80	98	7359.5	**<.001**	0.34
	Coerced	26	74	97.5	100	4857	**<.001**	0.53
Depression	Wanted	78.75	1	49	79.75	ref	ref	ref
	Inconsistent	59	37	72	96	11393.5	**<.001**	0.29
	Unwanted	48	50.75	81	98.75	7459	**<.001**	0.36
	Coerced	18.75	81.25	98	100	4977.5	**<.001**	0.57
Shame	Wanted	70	0	30.5	70	ref	ref	ref
	Inconsistent	44	51	79	95	12843.5	**<.001**	0.45
	Unwanted	54.25	42.75	76	97	7708	**<.001**	0.4
	Coerced	35.25	64.75	95	100	4895	**<.001**	0.54
Thoughts of child	Wanted	75.25	0.25	32	75.5	ref	ref	ref
	Inconsistent	54	36	72	90	11722	**<.001**	0.33
	Unwanted	57	36.75	75	93.75	7255	**<.001**	0.32
	Coerced	45.75	53.5	86	99.25	4516.5	**<.001**	0.42
Regret	Wanted	73.5	1	25.5	74.5	ref	ref	ref
	Inconsistent	57	34	71	91	11990	**<.001**	0.36
	Unwanted	66.5	30.5	77	97	7523.5	**<.001**	0.37
	Coerced	38	62	92	100	4822.5	**<.001**	0.52
Anxiety	Wanted	75.5	3	38	78.5	ref	ref	ref
	Inconsistent	57	35	68	92	11218	**<.001**	0.27
	Unwanted	57	40.75	77	97.75	7352	**<.001**	0.34
	Coerced	27.25	72.75	95.5	100	4923.5	**<.001**	0.55
Unforgiveness	Wanted	68	0	19	68	ref	ref	ref
	Inconsistent	56	38	69	94	12724.5	**<.001**	0.44
	Unwanted	52.25	44.75	78.5	97	7920.5	**<.001**	0.44
	Coerced	33.25	66.75	86.5	100	4883	**<.001**	0.54
Anger	Wanted	67.75	1	26.5	68.75	ref	ref	ref
	Inconsistent	50	29	61	79	11355	**<.001**	0.28
	Unwanted	64	33.75	74	97.75	7747.5	**<.001**	0.41
	Coerced	32	68	89	100	4954.5	**<.001**	0.56
Emptiness	Wanted	69.75	1	30	70.75	ref	ref	ref
	Inconsistent	48	36	64	84	11597	**<.001**	0.31
	Unwanted	55.5	40.25	72	95.75	7450.5	**<.001**	0.36
	Coerced	38.25	61.75	87.5	100	4805	**<.001**	0.51
Relief	Wanted	51.5	45.25	74	96.75	ref	ref	ref
	Inconsistent	54	23	50	77	6423	**<.001**	-0.027
	Unwanted	64.25	21.75	57.5	86	4543	**<.001**	-0.17
	Coerced	52.5	1	16.5	53.5	1533	**<.001**	-0.52
Difficult grieving	Wanted	61.75	0	19	61.75	ref	ref	ref
	Inconsistent	62	18	62	80	11561	**<.001**	0.31
	Unwanted	70.75	14.25	62.5	85	7307.5	**<.001**	0.33
	Coerced	55.75	44.25	81	100	4793.5	**<.001**	0.51
Weeping	Wanted	61.25	0	20.5	61.25	ref	ref	ref
	Inconsistent	68	13	63	81	11336.5	**<.001**	0.28
	Unwanted	75.75	17.25	69	93	7441.5	**<.001**	0.36
	Coerced	64.25	35	76	99.25	4676.5	**<.001**	0.47

Table 3 shows Mann-Whitney U statistics, p-values and Hedge’s g effect sizes for each emotion comparing each abortion decision type to pregnancy loss. The results show that, with the exception of shame, negative emotions were significantly less severe for women who had wanted abortions compared to women who had natural pregnancy losses with most differences being of moderate effect size (g > 0.3 and g < 0.5). For abortion decision types Inconsistent and Unwanted, the intensity of grief, depression, anxiety, emptiness, anger, weeping, thoughts of the child and difficulty completing the grief process were all similar (p > 0.5 and g < 0.1). But in the same group comparisons, feelings of guilt, regret, shame, and unforgiveness, all of which are related to self-judgment, were significantly more intense following an Inconsistent or Unwanted abortion with small to moderate effect sizes, Hedge’s g statistics ranging from 0.21 to 0.38. Similar findings were observed in the comparison of pregnancy loss to coerced abortions, but the effect sizes were stronger (g > 0.3) and the intensity of depression (g = 0.34), anxiety (g = 0.31), anger (g = 0.29), weeping (g = 0.18), and difficulty overcoming grief (g = 0.32) were also significantly and moderately greater for women who had coerced abortions. These findings support hypothesis H4 (decision types effect emotional reactions) and partially support hypothesis H3 which posited a similarity between natural pregnancy loss and abortion. H3 needs to be refined to reflect the effects of abortion decision types. Specifically, compared to natural pregnancy losses significantly fewer negative emotions are associated with wanted abortions and significantly more are associated with coerced abortions. The negative emotions associated with pregnancy loss are very similar to those following Inconsistent and Unwanted abortion decision types with the exception that additional negative emotions related to self-judgment appear with these abortions which are significantly less common among women with natural pregnancy losses.

**Table 3.** Mann-Whitney U statistics, p-values and Hedge’s g effect sizes for each emotion comparing the pregnancy loss group to each abortion decision type group.

	*Loss vs Wanted*			*Loss vs Inconsistent*			*Loss vs Unwanted*			*Loss vs Coerced*		
Emotion Scale	U	p	Hedge’s g	U	p	Hedge’s g	U	p	Hedge’s g	U	p	Hedge’s g
Grief	22454.5	**<.001**	-0.36	41666.5	0.96	0.003	25485.5	0.86	-0.01	18676	0.002	0.25
Depression	23796	**<.001**	-0.32	39876	0.45	-0.04	27275.5	0.38	0.06	19932	**<.001**	0.34
Anxiety	23496	**<.001**	-0.33	38470	0.17	-0.07	26413	0.71	0.02	19503	**<.001**	0.31
Guilt	30975.5	**0.05**	-0.11	52900	**<.001**	0.27	32637.5	**<.001**	0.27	20617	**<.001**	0.38
Relief	51040.5	**<.001**	0.46	53219	**<.001**	0.28	34587	**<.001**	0.34	13911	0.43	-0.07
Emptiness	19984	**<.001**	-0.43	34984.5	0.003	-0.16	23849.5	0.25	-0.08	17259	0.06	0.16
Anger	22841.5	**<.001**	-0.35	37990.5	0.11	-0.09	28364	0.13	0.1	19126.5	**<.001**	0.28
Regret	29215	**0.004**	-0.16	50363.5	**<.001**	0.21	31959	**<.001**	0.24	21087.5	**<.001**	0.42
Shame	31662	0.10	-0.09	57306	**<.001**	0.38	34276	**<.001**	0.33	22131.5	**<.001**	0.49
Unforgiveness	27075	**<.001**	-0.23	51096.5	**<.001**	0.23	32444.5	**<.001**	0.26	20668	**<.001**	0.39
Weeping	22632.5	**<.001**	-0.35	37668.5	0.08	-0.09	26432	0.70	0.03	17558.5	**0.03**	0.18
Thoughts of child	23364.5	**<.001**	-0.33	40165	0.54	-0.03	25424	0.83	-0.01	17013.5	0.09	0.14
Difficult grieving	26616.5	**<.001**	-0.24	44397.5	0.20	0.07	28341.5	0.13	0.1	19735.5	**<.001**	0.32

Table 4 (Extended data) shows the emotion intensity scales ranked from highest to lowest median for each group with Wilcoxon signed rank statistics for each emotion compared to Relief within each group. The results show that Relief was the most strongly felt emotion only among women whose abortions were wanted and consistent with their values and preferences. The effect size was moderate (RBC > 0.3) to large (RBC > 0.5) for most comparisons. For all other abortion decision types, Relief was the least strongly felt emotion. All negative emotions were significantly greater than Relief for the Coerced and Pregnancy Loss groups. For the Unwanted group, only guilt, depression and grief were significantly greater than relief, though many of the other negative reactions approached significance and appear likely to be significant with a larger sample size. Effect sizes varied within the groups. For example, in the Inconsistent group, there was only a small difference in the intensity of anxiety (RBC = -0.29) compared to relief but a moderate difference in depression (RBC = -0.37) and large degrees of difference relative to shame (RBC = -0.52) and guilt (RBC = -0.63). The differences within the Coerced group were all very large, with RBC < -0.7). These results suggest rejecting the null hypothesis, H1, except for women whose abortions are freely chosen and are consistent with their values and preferences. The results also support hypotheses H2 and H4, positing that negative emotions are more prominent than relief, especially when the abortion decision is not aligned with women’s own values and preferences.

A linear regression analysis was performed using the scale grading the degree of stress experienced in completing the survey as the dependent variable with all thirteen emotion variables and abortion decision types as covariates. The model was statistically significant, F(16, 392) = 8.40, p < .001, and explained 22% of the variance in survey taking stress levels (Adjusted R ^2^ = 0.22). Unstandardized (B) and standardized (β) coefficients are shown in Table 5. Anxiety (β = 0.22, p = .006) and anger (β = 0.14, p = .04) significantly increased stress levels. Additionally, women in the Inconsistent (B = 8.43, p = .03) and Coerced (B = 10.82, p = .05) decision type groups reported significantly higher stress when completing the survey. These findings support hypothesis H5, which posited that more negative feelings would be associated with greater stress in completing surveys about the abortion experience.

**Table 5.** Selected covariates for greater stress completing the abortion history survey.

Covariate	B	S.E.	β ^a^	t	p
Grief	-0.07	0.08	-0.08	-0.88	0.38
Depression	-0.14	0.09	-0.15	-1.66	0.10
Anxiety	0.2	0.07	0.22	2.75	**<0.01**
Guilt	0.04	0.07	0.04	0.54	0.59
Relief	-0.03	0.05	-0.03	-0.71	0.48
Emptiness	0.09	0.07	0.1	1.25	0.21
Anger	0.13	0.07	0.14	2.03	**0.04**
Regret	-0.05	0.07	-0.05	-0.67	0.50
Shame	0.07	0.07	0.08	1.02	0.31
Unforgiveness	0.06	0.07	0.06	0.8	0.42
Weeping	0.05	0.06	0.05	0.73	0.47
Thoughts of child	-0.02	0.06	-0.02	-0.36	0.72
Difficult grieving	0.11	0.07	0.12	1.66	0.10
Inconsistent	8.43	3.97	na	2.12	**0.03**
Unwanted	6.96	4.38	na	1.59	0.11
Coerced	10.82	5.47	na	1.98	**0.05**

^a^ Standardized coefficients can only be computed for continuous predictors.

## Discussion

These findings challenge the assertion that relief is the most common emotional reaction to abortion, as previously suggested by studies with high attrition rates and potential selection biases. ^11, 13^ Notably, our findings align with previous research indicating that women who experience pressure to abort or feel conflicted about their decision are more likely to report enduring negative emotions. ^6, 7^ The presence of these negative feelings suggests that the psychological impact of abortion is complex and cannot be generalized as a uniformly positive or neutral experience.

One of the key findings of this study is the strong association between abortion decision type and emotional outcomes. Women who classified their abortions as coerced reported significantly more distressing emotions than those who described their abortions as fully wanted. The intensity of negative emotions among women in the coerced and unwanted abortion groups suggests that external pressures and a lack of personal autonomy in decision-making may contribute to psychological distress.

### Hypotheses tested

The null hypothesis, H1, posited that relief is the most common emotional reaction to abortion. Our findings require rejection of this hypothesis. On average, for the grouping of all women reporting a history of abortion, relief is neither the most frequently reported nor dominant emotion attributed to their abortion experiences. Instead, grief, guilt, shame, depression and regret were all more prevalent and dominant (Table 4).

Hypothesis H2 posited that negative emotions would be more common than relief. This was confirmed in all group comparisons except for women whose abortions were wanted and consistent with their values and preferences. Wanted is the only subgroup reporting relief as more common and dominant than negative emotions (Table 4). Reports from other studies finding that relief is the most dominant reaction to abortion are probably best explained by methodological and self-selection biases which result in samples that underrepresent women in the abortion decision types Inconsistent, Unwanted, and Coerced.

Hypothesis H3 posited that the emotional impact of abortion would be similar to that of natural pregnancy loss. Our comparison between all women with a history of abortion and those with natural losses ( Table 1) revealed several similarities and relatively small differences in effect sizes. However, when the abortion group were disaggregated into decision types, pregnancy loss emotions are very dissimilar to wanted abortions but more similar to Inconsistent, Unwanted and Coerced decision types ( Table 3).

Hypothesis H4 proposed that the degree of negative emotions following an abortion would vary based on how consistent the decision was with a woman’s values and preferences. This hypothesis is strongly supported by the data ( Tables 2 and 4), which demonstrate that Coerced, Unwanted and Inconsistent abortion decision types were associated with more intense negative emotions, all of which were rated above that of relief, whereas Wanted abortions were more frequently associated with relief as more predominant than negative emotions.

Hypothesis H5 proposed that negative emotions would be associated with higher stress levels when participating in surveys about abortion experiences. The data confirm this hypothesis, as women who reported strong negative emotions, particularly those who categorized their abortion as coerced, also experienced higher stress levels when completing the survey. These findings are also consistent with the studies indicating that women who anticipate or are experiencing more negative feelings are more likely to dropout or to decline to participate in post-abortion surveys. ^17, 18^

### Strengths and limitations

A major strength of this survey is that the proportion of women reporting a history of abortion closely aligns with the Guttmacher Institute’s estimates for lifetime abortion exposure rates in this age group. ^29^ While most national surveys capture less than half of the expected abortion rates, ^14, 15^ our sample was only 10.5% below the Guttmacher Institute’s estimate—which is itself only an estimate based on voluntarily provided abortion clinic data. Given that underreporting of abortion histories in surveys is often linked to feelings of shame and other negative reactions, ^17, 18^ it is likely that undisclosed abortion experiences would lead to an underestimation rather than an exaggeration of the negative emotional reactions reported here. The higher rate of abortion disclosure in this survey may be attributed to the inclusion of an experimental stigma reduction protocol. It may also be due to the use of Cint survey panels which include women who are experienced at completing surveys and may have a higher trust and tolerance for anonymously reporting sensitive information. Women aged 41-45 may also be more willing to share information about past abortion experiences, perhaps because the laps of many years has contributed to moderation or healing that a younger group of women have not yet experienced.

An additional strength is that our survey minimized selection bias by recruiting from a national survey panel rather than abortion clinics. Moreover, while there are likely significant demographic variations in our sample from national averages, the sampling process was random and relationships observed between the study groups are likely to hold across different sampling methodologies. It is unlikely that any underlying factors that lead some women to participate in Cint survey panels more than others are themselves strongly biased toward different emotional reactions to abortion and pregnancy loss.

Another significant strength of this study is its novel examination of emotional responses segregated by different abortion decision types. The strong and important differences in emotional responses observed across different abortion decision types suggest that these decision types should be included in all abortion mental health research. Finally, another strength is that this survey design can be readily and cheaply replicated and improved upon.

Despite these strengths, this study has several limitations. First, it is entirely retrospective. Memories and emotions may have changed over time. Responses given in hindsight could differ from those that might have been reported shortly after the pregnancy outcomes examined in the survey. On the other hand, emotions attributed to pregnancy loss at any time are relevant to patients and counselors. While it would be helpful to better identify the relative strength of emotions throughout women’s lifetimes, these findings regarding women aged 41-45 are relevant and important in their own right.

A second, similar limitation, concerns the timing of emotional reactions. Due to questionnaire length constraints, the study did not capture the precise onset and duration of each reported emotion. Future research should incorporate both retrospective and longitudinal approaches to address these gaps.

Additionally, 56.3% of the women in the abortion group also reported a history of pregnancy loss. It is possible that their combined experiences of abortion and natural pregnancy loss influenced their responses. Indeed, it is well known that a subsequent pregnancy loss may trigger suppressed issues associated with a prior abortion. ^5, 8, 30^ Further research is needed to disentangle these overlapping experiences and identify other contributing factors that may shape post-abortion emotional responses.

While this study used a large, randomly selected sample, the absence of data on women younger than 41 years means the findings may not be fully generalizable to younger populations. Additional research is needed to investigate younger groups of women and provide grading of emotions closer to the abortion and pregnancy loss experiences.

### Recommendations

Women considering abortion should not be told that relief is the most common reaction to abortion. Abortion counselors should screen patients for abortion decision type and the risk factors identified by the American Psychological Association’s Task Force on Mental Health and Abortion. ^12, 28^ Mental health workers should be aware of these risk factors and the different abortion decision types. Women seeking mental health care should be routinely asked if they have experienced any “pregnancy losses, like miscarriage, abortion, or a stillbirth” as a means of inviting them to discuss any lingering negative emotions. In the experience of some pregnancy loss counselors, many clients are prone to hold their abortion experiences as tight secrets. An explicit invitation to discuss any history of abortion or pregnancy losses may free them to share what they have never discussed before, and thereby, open the door to emotional healing. ^5^

## Conclusion

Overall, these findings strongly refute the claim that for 95% of abortion patients their most dominant feeling will be relief. These findings also highlight the importance of abortion decision type on post-abortion emotional outcomes. The presence of significant negative emotions among women whose abortions conflicted with their own values suggests the need for greater attention to pre-abortion screening, informed consent, and referrals for support services. Policies and counseling approaches should acknowledge the diversity of post-abortion emotions and provide appropriate psychological support, particularly for women who feel pressured into abortion or who experience significant emotional distress afterward. More research, resources and training should be directed toward improving the effectiveness and availability of post-abortion counseling for those who experience distress.

### Ethical review and informed consent

All participants were adults who provided electronically signed informed consent to complete a survey that might address sensitive issues. No personal identifying information was collected. All procedures contributing to this work comply with the ethical standards of the relevant national and institutional committees on human experimentation and with the Helsinki Declaration of 1975, as revised in 2008, and were approved by Sterling Institutional Review Board issued (ID:10225; July 18, 2022) which determined that under U.S. Department of Health and Human Services Policy for Protection of Human Research Subjects at C.F.R. 46.101(d) this study regiman is exempt from further review since it relies on surveys where the subjects’ identities are protected and disclosures of the anonymous information collected pose no risks to the participants.

## Data Availability

Zenodo: A Survey of Emotional and Mental Health Effects Women Attribute to Their Pregnancy Outcomes. https://doi.org/10.5281/zenodo.13941640. ^31^ This project contains the following underlying data:
• Survey w calculated fields.csv: anonymized responses to all survey items, plus a variety of constructed summary variables from those responses. Data are available under the terms of “ https://creativecommons.org/licenses/by-nc/4.0/deed.en” Creative Commons Attribution-Noncommercial 4.0 International license (CC-BY-NC 4.0). Zenodo: A Survey of Emotional and Mental Health Effects Women Attribute to Their Pregnancy Outcomes. https://doi.org/10.5281/zenodo.13941640. ^31^ This project contains the following underlying data:
• 2nd USA survey limesurvey.lss: the LimeSurvey format survey structure which can ben run using LimeSurvey software. • 2nd USA Survey Instrument in Word.docx: the survey questions and structure outputted to a Word document. • Cint Consent.pdf: The consent forms and user use agreements. • Relief Results v4 Rp.xlsx: Large table results published in this paper which includes Table 4. • Respondents Flochart.png Data are available under the terms of “ https://creativecommons.org/licenses/by-nc/4.0/deed.en” Creative Commons Attribution-Noncommercial 4.0 International license (CC-BY-NC 4.0).

## Software availability statement

LimeSurvey is licensed under the GNU General Public License (GPL) and available from LiveSurvey.org at https://community.limesurvey.org/downloads/.
