# Supplementary material for: Is relief the most common reaction to abortion? Self-assessed intensity of emotions attributed to abortion in a national sample of women aged 41 to 45
Source: F1000Res. 2025 Apr 3;14:240. Originally published 2025 Feb 26. [Version 2] doi: 10.12688/f1000research.162063.2 (PMC11933783; doi:10.12688/f1000research.162063.2)
Supplement: Peer review history

# Reviewer report: Reviewer response for version 1

**DOI:** 10.5256/f1000research.178189.r368871

**Published:** 2025-03-24

**Authors:** Priscilla Coleman

**Affiliations:** 1 International Institute for Reproductive Loss, Brooklyn, USA

This article clarifies a misrepresentation related to the post-abortion relief construct that has permeated society generally and professional organizations in particular. The methodology is appropriate, strong, and described effectively. The results are compelling, given the sample size and representativeness, comparison groups, and the sensitivity of the outcome response scales.                 

 I suggest placing the description of the results relative to the hypotheses tested under the "Results" section rather than under the "Discussion" section. Please provide more information on how the experts developed the questionnaire. Finally, this article is a single-author publication, yet the first-person plural was used in the manuscript. Adjustments should be made to correct this inconsistency.

Is the work clearly and accurately presented and does it cite the current literature?

Yes

If applicable, is the statistical analysis and its interpretation appropriate?

Yes

Are all the source data underlying the results available to ensure full reproducibility?

Yes

Is the study design appropriate and is the work technically sound?

Yes

Are the conclusions drawn adequately supported by the results?

Yes

Are sufficient details of methods and analysis provided to allow replication by others?

Yes

Reviewer Expertise:

The psychology of abortion: decision-making and mental health trajectories/outcomes; Early socioemotional development, especially attachment; Transition to parenting: parenting cognitions, particularly self-efficacy; Loss to the birth mother through adoption.

I confirm that I have read this submission and believe that I have an appropriate level of expertise to confirm that it is of an acceptable scientific standard.

# Response

**Published:** 2025-03-24

**Authors:** David Reardon

**Affiliations:** Elliot Institute, Gulf Breeze, Florida, USA

Thank you for your review and recommendations.

 I decided to keep the discussion of the tested hypotheses in the discussion section. I believe it is best to review the results in context of the hypotheses tested in this section, especially for readers who may skim past both the introduction and results, heading straight for the summary of findings in the discussion section.

 I did fix all the plural self references, as you suggested.

 Thank you.

# Reviewer report: Reviewer response for version 1

**DOI:** 10.5256/f1000research.178189.r369990

**Published:** 2025-03-20

**Authors:** Walter Schumm

**Affiliations:** 1 Kansas State University, Manhattan, Kansas, USA

1.  The literature review does not appear to cite articles published in the last five years very often, so that could be improved.

 2.  It would be helpful if the dropouts could be compared to those who responded.  Agencies that collect data as used here often have baseline demographics for their panel participants which might be made available to permit comparisons of dropouts and respondents.

 3.  I know of a personal case where Dr. Tiller (who was later murdered) persuaded a potential client to not get an abortion, so it's possible for abortion providers to screen their clients carefully and not just insist on abortions for all who discuss it with them.

 4.  The most serious flaw here seems to this reviewer to be that basic demographic variables are not reported or used as controls.  Education might be the most important of these. 

 5.  A limitation is that the ages of the women when they had their birth/abortion experiences are not accounted for.

 6.  The protocols would seem to hinder an analysis of how combinations of birth/abortion experiences lead to various outcomes.  For example, are apparent reactions different for women who have an abortion and then do or do not have later natural births?  Are reactions different if a woman has an abortion and miscarriages versus not having any later or previous miscarriages?  Sample sizes might be too small for analysis of some combinations, of course. 

 7.  While I don't see it as necessary here, use of more complex forms of analysis might be useful.

 8.  In Table 1, when Hedge's g is reported at .1 it should be .10 to be consistent with how others are reported.

Is the work clearly and accurately presented and does it cite the current literature?

Partly

If applicable, is the statistical analysis and its interpretation appropriate?

Yes

Are all the source data underlying the results available to ensure full reproducibility?

Yes

Is the study design appropriate and is the work technically sound?

Yes

Are the conclusions drawn adequately supported by the results?

Yes

Are sufficient details of methods and analysis provided to allow replication by others?

Yes

Reviewer Expertise:

Methodological evaluations of controversial research in areas of human sexuality

I confirm that I have read this submission and believe that I have an appropriate level of expertise to confirm that it is of an acceptable scientific standard.

# Response

**Published:** 2025-03-24

**Authors:** David Reardon

**Affiliations:** Elliot Institute, Gulf Breeze, Florida, USA

I have expanded the discussion of limitations to address the limitations you have raised.

 I agree with the point 6, in which you note how there may be additional differences related to different pregnancy outcome patterns. It is my hope to address this in future research.

 Additionally, while not noted in the revision, your interest in a comparison of dropouts was at least partially addressed in the first paragraph of the results section in Reardon D C, Longbons T (January 31, 2023) Effects of Pressure to Abort on Women’s Emotional Responses and Mental Health. Cureus 15(1): e34456. doi: 10.7759/cureus.34456. While that study did not include all of the same questions, it used the same Cint panel criteria and stigma reduction questions. In that study, "Women with a history of abortion were over four times (odds ratio (OR)=4.43, 95% confidence interval (CI)=2.31-8.49) more likely to drop out of the survey at or after the first question related to abortion compared to women who did not report a history of abortion and were routed to a different set of questions regarding their reproductive lives."  I would presume there are somewhat similar self-censure effects in the present study.
